# Nanotextured Shrink Wrap Superhydrophobic Surfaces by Argon Plasma Etching

**DOI:** 10.3390/ma9030196

**Published:** 2016-03-14

**Authors:** Jolie M. Nokes, Himanshu Sharma, Roger Tu, Monica Y. Kim, Michael Chu, Ali Siddiqui, Michelle Khine

**Affiliations:** 1 Department of Biomedical Engineering, Samueli School of Engineering, University of California, Irvine; Irvine, CA 92697, USA; mclanej@uci.edu (J.M.N.); monicayk@uci.edu (M.Y.K.); mchu8@uci.edu (M.C.); siddiqa1@uci.edu (A.S.); 2 Department of Chemical Engineering and Material Sciences, Samueli School of Engineering, University of California, Irvine; Irvine, CA 92697, USA; sharmah@uci.edu; 3 Department of Biology, Ayala School of Biological Sciences, University of California, Irvine; Irvine, CA 92697, USA; tur@uci.edu

**Keywords:** bioinspired material, argon plasma treatment, superhydrophobic, protein capture, detection, wicking, microfluidics, shrink film, fabrication

## Abstract

We present a rapid, simple, and scalable approach to achieve superhydrophobic (SH) substrates directly in commodity shrink wrap film utilizing Argon (Ar) plasma. Ar plasma treatment creates a stiff skin layer on the surface of the shrink film. When the film shrinks, the mismatch in stiffness between the stiff skin layer and bulk shrink film causes the formation of multiscale hierarchical wrinkles with nano-textured features. Scanning electron microscopy (SEM) images confirm the presence of these biomimetic structures. Contact angle (CA) and contact angle hysteresis (CAH) measurements, respectively, defined as values greater than 150° and less than 10°, verified the SH nature of the substrates. Furthermore, we demonstrate the ability to reliably pattern hydrophilic regions onto the SH substrates, allowing precise capture and detection of proteins in urine. Finally, we achieved self-driven microfluidics via patterning contrasting superhydrophilic microchannels on the SH Ar substrates to induce flow for biosensing.

## 1. Introduction

Superhydrophobic (SH) surfaces are found widely throughout nature with a variety of purposes. For example, the lotus leaf consists of micro and nano-structured features that allows for self-cleaning properties, while the Namib Desert beetle has alternating SH and hydrophilic regions to optimize water collection under extreme desert conditions [1,2,3]. Inspired by these naturally occurring SH surfaces, both industrial and academic sectors have significant interest in mimicking and recreating the unique water-repellant property of SH features for numerous commercial and biomedical applications [4]. In particular, commercial applications include creating surfaces that are self-cleaning, desalinating, anti-corroding, anti-icing, or anti-bacterial. On the other hand, potential biomedical applications can encompass enhanced biosensing, point-of-care detection, drag reduction in microfluidic devices, and delayed coagulation in blood [5,6,7,8,9,10]. 

SH surfaces are substrates with low surface energy, which significantly decreases the adhesion of water. Superhydrophobicity is characterized as having a water contact angle (CA) greater than 150° and a contact angle hysteresis (CAH) less than 10° [11,12,13]. Engineered SH surfaces can be attained through adding structural or chemical modifications to a material [14,15,16,17]. Structural SH surfaces can be created when hierarchal micro-nanoscale structures are incorporated onto inherently hydrophobic surfaces [18]. These structures robustly trap small pockets of air underneath a water droplet, resulting in a high contact angle, low contact angle hysteresis, and weak adhesive force, known as the Cassie-Baxter model [12,19]. According to this model, the water droplet sits on the peaks of the structures with air pockets trapped between the water droplet and the surface, causing a weak adhesion between the substrate and fluid. 

Methods to fabricate SH surfaces include depositing material with low surface energy onto a rough surface, roughening a hydrophobic surface via etching, creating hierarchal architectures on a substrate, or a combination of these techniques [20,21,22,23]. However, these approaches typically require additional post-processing using chemicals to decrease the surface energy [11,24,25]. Chemical modifications can cause health and environmental concerns due to the possibility of chemical leaching [26]. Additionally, chemical modification can wear off over time, and the SH nature can diminish. Due to these limitations, SH surfaces manufactured solely with structural modifications are more desirable than those utilizing chemical modifications. 

In our previous work, we achieved purely structural SH surfaces by shrinking pre-stressed thermoplastic polymers with metal thin films deposited on top, resulting in multi-scale wrinkled structures. These hierarchical, biomimetic structures could subsequently be transferred to a silicone elastomer mold, which yielded a SH elastomeric substrate [7,27,28]. The structures can further be hot embossed into inherently hydrophobic materials, such as commodity plastics, for a variety of applications. 

Here, we present a simple, inexpensive method to create structural SH surfaces that obviates the need for metal deposition or multiple feature transplantations. In this approach, multi-scale wrinkled SH structures were created directly in commodity shrink film by simply treating the film with Ar plasma and subsequently heating the substrate past its glass transition temperature to shrink it. We demonstrate feasibility of patterning the SH substrates with hydrophilic regions to capture protein in urine with a wide range of detectable protein concentrations. Furthermore, open-channel, self-wicking microfluidic flow can also be achieved via patterning superhydrophilic regions on the SH substrates. This drives fluid flow due to a high surface energy differential accomplished between a SH and superhydrophilic interface. 

## 2. Materials and Methods

### 2.1. Fabrication of SH Surfaces via Ar Gas Plasma Treatment

A 4.5” × 4.5” polyolefin (PO) film (seven layer polyethylene/polypropylene blend, Sealed Air Corp., Renton, WA, USA) was cut and clamped securely onto a 5” × 5” glass slide and placed into a plasma cleaning system (South Bay Technology Plasma Cleaner PC-2000, South Bay Technology Inc., San Clemente, CA, USA). The PO film was plasma treated with Ar for 1, 5, 10, 20, 30, 40, and 60 min (at 200 mTorr and 60 W). After plasma treatment, the PO films were shrunk to induce the multiscale, hierarchical wrinkling. Briefly, the PO films were sandwiched between two glass slides and two sheets of parchment paper with 1 mm rubber spacers. Four 0.5” binder clips were added to apply pressure to the sandwich. The films were then heated to 120 °C in a conventional convection oven (Black & Decker TO3250XSB Toaster Oven, Black & Decker, Townson, MD, USA), allowing the PO film to shrink within the constraints of the glass slides.

### 2.2. Surface Characterization

The surface topology of the shrunk Ar-treated substrates were observed using a high-resolution scanning electron microscope (SEM) (FEI Magellan 400 XHR SEM, FEI, Hillsboro, OR, USA). Prior to imaging, 4 nm of iridium was sputtered on top of the substrates to prevent charging. For each sample, the distribution of wrinkle wavenumber, defined as the inverse wavelength, was found by applying a 2D Fast Fourier Transform (2D FFT) on the SEM images using Matlab^®^ (MathWorks Inc. R2015a, Natick, MA, USA) [28,29]. The absolute value of the 2D FFT output was centered at 0 wavenumber. The wavelength distribution was calculated by integrating the intensities radially for each wavenumber, resulting in a plot of intensity *versus* wavenumber. A running average of the intensity and wave number was taken, and each plot was normalized to its maximum value. The average noise summed for each wavenumber was estimated and subtracted out [28,29]. 

Additionally, roughness measurements were taken using a red laser scanning microscope (Keyence VK-X100, Keyence, Osaka, Japan). The root mean square (RMS) was taken to compare microscale roughness of the features on the shrunk surfaces for different treatment times. Experimentally, such optical measurements using our imaging system are limited in resolution compared to a scanning microscope and, thus, microscale rougness resolution is comparable to FFT data corresponding to 5 µm and greater (*i.e.*, cannot definitely determine submicron features). Therefore, coupled with the nanoscale FFT measurements, microscale roughness data from the laser scanning microscope was used to characterize the SH nature of our substrates.

### 2.3. Contact Angle and Contact Angle Hysteresis

Contact angle (CA) and contact angle hysteresis (CAH) measurements were taken using a droplet shape analyzer (DSA-30, KRÜSS Optronic GmbH, Hamburg, Germany). For the CA measurements, shrunk Ar-treated samples were cut and mounted onto standard microscope slides with double-sided tape. The samples were placed on the DSA-30, and CA values of 5 μL of water were measured via tangent analysis with ADVANCE software (KRÜSS ADVANCE v1.2, KRÜSS Optronic GmbH, Hamburg, Germany). The mean CA for each independent substrate was calculated by averaging the CA over three individual spots on each sample.

CAH values were evaluated by depositing 10 µL of water at a constant rate of 0.16 µL·min^−1^ to record advancing CA. The droplet was then retracted at a constant rate of 0.16 µL·min^−1^ to measure receding CA. CAH was calculated by taking the difference of advancing and receding CA [13,30]. The mean CAH for each independent substrate was also calculated by averaging over three individual spots on each sample.

### 2.4. Hydrophilic Patterning

Non-Ar-treated PO film was shrunk in the same manner as the Ar-treated films as a control surface. The non-Ar-treated substrates and the SH Ar-treated substrates were patterned with hydrophilic regions using a negative mask and air gas plasma treatment. A mask with 2, 1, and 0.5 mm holes (with 5 mm spacing) was created by cutting the design into a paraffin film substrate (Parafilm-M^®^ Laboratory Sealing Film, Bemis Co., Neenah, WI, USA) with a CO_2_ laser (Universal Versa Laser Systems 2.30, Universal Laser Systems, Scottsdale, AZ, USA). The laser cut Parafilm mask was adhered to a non-Ar-treated substrate or a SH Ar-treated substrate by applying minimal pressure. The substrates were subsequently treated with air plasma (Plasma Etch PE-50 XL, Structure Probe Inc., West Chester, PA, USA) for 1 min at 75% power to achieve hydrophilic anchor points. Droplets of diluted green food dye (30, 15, and 5 µL, Kroger Food Colors, Kroger, Cincinnati, OH, USA) were deposited on the patterned substrates (2, 1, and 0.5 mm spots, respectively). Top view and side view images were taken with a DLSR camera (Canon EOS 5D, Canon USA Inc., Melville, NY, USA) using a 100 mm lens (Canon Macro Lens EF 100 mm, Canon USA Inc., Melville, NY, USA).

### 2.5. Protein Capture and Detection

A standard colorimetric Bradford protein assay (Bio-Rad Protein Assay) was used to detect protein concentrations. Urine was collected from voluntary subjects (in accordance with UC Irvine Institutional Review Board—Health Sciences #9012-9022), and the baseline urine protein concentration was calculated using absorbance values from a ultraviolet-visible (UV-VIS) spectrophotometer (Thermo Scientific Nanodrop 2000C Spectrophotometer, NanoDrop products, Wilmington, DE, USA) compared to a standard curve derived with bovine serum albumin (BSA) (Sigma-Aldrich, St. Louis, MO, USA). Urine samples were then diluted with DI (deionized water) water or spiked with BSA to achieve final protein concentrations of 0, 20, 40, 80, 150, 200, and 250 µg·mL^−1^. Using a Parafilm mask and air plasma treatment (Plasma Etch PE-50 XL, Plasma Etch, Carson City, NV, USA) at 1 min, 75% power respectively, a 3 × 3 array of 500 µm diameter hydrophilic spots with 5 mm spacing was patterned onto the SH Ar substrates. 10 µL of adjusted urine was deposited on each spot and allowed to evaporate for 2 h. After evaporation, 10 µL of diluted protein assay dye reagent was added and incubated for 5 min. The reactants were then removed from the substrates, and the absorbance values were measured at 595 nm on the Nanodrop. Reported values are the signal minus the 0 µg·mL^−1^ control.

### 2.6. Microfluidic Wicking in Superhydrophilic Channels

Similar to the patterning and protein detection, a laser cut Parafilm mask and air plasma treatment (Plasma Etch PE-50 XL, 3 min and 75% power) was used to create self-driven, open-channel microfluidic flow. The mask consisted of 400 µm wide channels. To demonstrate self-wicking, 5 µL of diluted green food dye was added to the inlet of the microchannel, and self-driven fluid flow was recorded using a high speed camera (Sony Alpha 7s, Sony, Tokyo, Japan) at 120 fps and a macro lens (Canon Macro Photo MP-E 65 mm, Canon USA Inc., Melville, NY, USA). 

## 3. Results and Discussion

### 3.1. Nanotextured Superhydrophobic Wrinkle Topology

**Figure 1:**
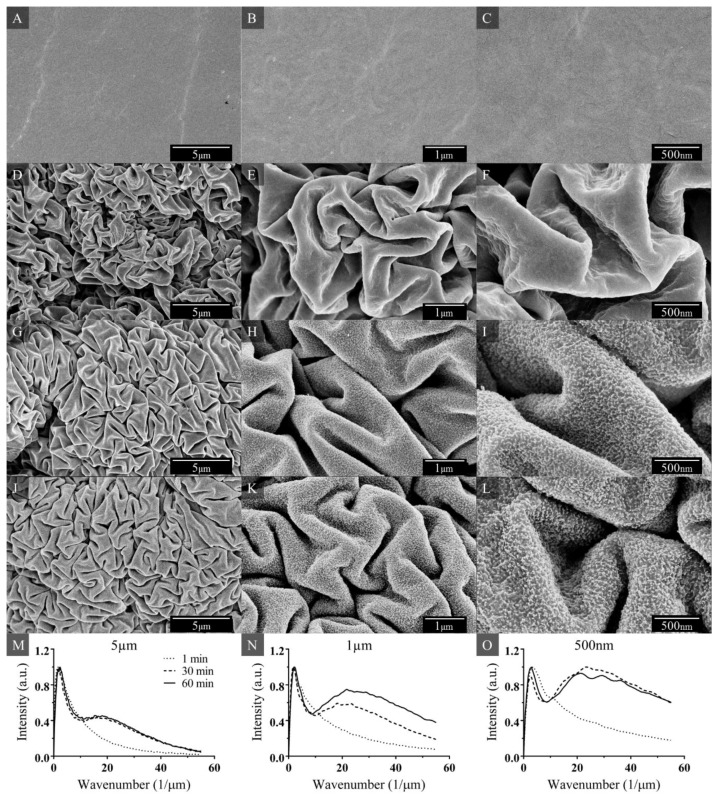
SEM images of shrunk surfaces that were not Ar-treated (**A**–**C**); and Ar-treated for (**D**–**F**) 1 min; (**G**–**I**) 30 min; and (**J**–**L**) 60 min. FFT graphs showing spatial frequency of the hierarchical features for shrunk Ar-treated samples at 1, 30, and 60 min (**M**–**O**).

SEM images of the surface morphology of the non-Ar-treated and Ar plasma treated (1, 30, 60 min) shrunk substrates are shown in Figure 1. As expected and observed in Figure 1A–C, the shrunk surface was relatively smooth and flat on the non-Ar-treated shrunk PO film. In contrast, when the PO film was Ar-treated for 1 min, multiscale wrinkled features were formed (Figure 1D–F). These multiscale wrinkles were also formed when the PO film was Ar-treated for 30 min (Figure 1G–I) and 60 min (Figure 1J–L). 

The wrinkle formation was attributed to the stiff skin layer created on the surface of the pre-stressed PO film due to the Ar plasma treatment. During plasma treatment with the inert Ar gas, energized ions from the bulk plasma break C-H or C-C bonds on the surface to create free radicals on the surface of the PO. When the Ar-treated PO is removed from the chamber and exposed to air, the free radicals react with the molecular oxygen to form a stiff oxide layer on the PO surface [31,32]. When the treated substrate was heated past the glass transition temperature to induce shrinking, the stiffness mismatch between the skin and PO film resulted in buckling and hierarchical wrinkled features [33]. A similar process was previously applied and described on pre-stressed polystyrene substrates by Huntington *et al.* [29,34]. Similarly, Stenberg *et al.* reported wrinkling due to the stiff skin layer developed by argon plasma treatment on their polystyrene (PS) samples [35]. 

The zoomed out SEM images in Figure 1D (1 min Ar treatment) and 1G (30 min Ar treatment) reveal that the multiscale wrinkles appear to be of a similar wavelength despite the difference in plasma exposure time. From buckling theory, it is well understood that the wavelength of the wrinkles is linearly proportional to the skin thickness and to the cube root of the ratio between the elastic modulus of the skin layer and substrate. The SEM images reveal that the wavelength of the wrinkles do not appear to change significantly with longer plasma treatments, suggesting that the elastic modulus of the skin thickness and/or film is not altered by variable plasma treatment time. 

Accordingly, Bruce *et al.* noted that when certain polymers were exposed to Ar plasma, a dehydrogenated amphorous carbon layer formed on the surface, and the thickness remained constant once the initial layer was formed [9]. Interestingly though, the magnified SEM image of 30 min Ar exposure (Figure 1I) reveals small nanoscale features on the wrinkles that are visibly absent in the 1 min Ar exposure sample (Figure 1F). Similar nanoscale features are observed for the 60 min treatment as seen in Figure 1J–L. When comparing the magnified SEM images of the 30 min (Figure 1I) *versus* the 60 min (Figure 1L) Ar treatment, the nanoscale roughness appears to be more pronounced with the longer exposure time. 

Wrinkle wavelength and nanofeature roughness were characterized further by applying 2D FFT to the SEM images. The 2D FFT algorithm was applied to each SEM image in Figure 1D–L, and the resulting plots for each exposure time were graphed together for each length scale. As expected from our previous visual assessment, we observed only one distinct peak for the one-minute treated surface, in contrast to two distinct peaks for surfaces treated at 30 and 60 min. Treatment at 1, 30, and 60 min all produced larger wrinkles ranging from 300 to 600 nm, while the second peak in the distribution for the 30 and 60 min treatment was between 40 to 60 nm. 

**Figure 2:**
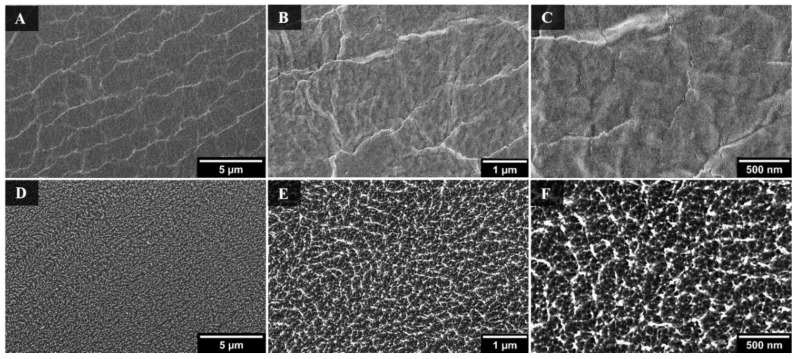
SEM images of flat PO film without Ar treatment (**A**–**C**); and with 30 min Ar treatment (**D**–**F**).

To develop a better understanding of the nanoscale features observed on the multiscale wrinkles, SEM images of the PO film were captured prior to shrinking on a non-Ar-treated *versus* an Ar-treated (30 min) sample, as shown in Figure 2. These images reveal that continuous Ar plasma treatment of the surfaces results in significant etching to the plastic film and yields a highly roughened surface relative to the untreated PO film. This phenomenon has been previously described by Bruce *et al.* where Ar plasma was used to induce nanoscale roughness on polystyrene films [36]. However, the nanoscale features observed on our shrunk, multiscale wrinkled surfaces were not present on their nanostructured surfaces due to our pronounced shrinking method.

In addition to wrinkle wavelength and nanoscale roughness, microscale roughness was also characterized because a combination of nanoscale and microscale features are necessary to achieve superhydrophobicity. The microscale features on the Ar-treated SH samples were observed using a red laser scanning microscrope, as shown in Supplemental Figure S1. Due to the experimental limitations of our optical imaging system, nanoscale features could not be observed. The microscale roughness measurements collected from the laser scanning microscope were experimentally comparable to the microscale region of the FFT data presented in Figure 1M. 

Samples treated with Ar for 1, 30, and 60 min result in microscale roughness of 2.2 ± 0.4 µm, 4.4 ± 0.6 µm, and 5.0 ± 0.2 µm, respectively. As plasma treatment increases, the microscale roughness increases, which could be attributed to the effective skin on our shrink film [28]. Under high compression (such as without the PO film), hierarchical wrinkles occur because the amplitude of the smaller, first generation wrinkles saturate and create an effective skin. This effective skin in turn generates an effective generation of wrinkles. Therefore, samples that were Ar plasma treated for 30 and 60 min have a greater microscale roughness because the nanoscale features shown in Figure 2 create an effective skin that affects the wrinkling of the second generation wrinkles. This increased microscale roughness aids in superhydrophobicity for samples Ar plasma treated for longer periods of time. 

### 3.2. Superhydrophobicity

**Figure 3:**
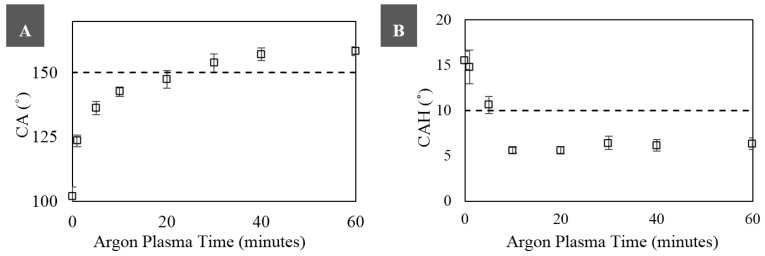
Longer Ar plasma treatment times correspond to superhydrophobicity. (**A**) Water CA; and (**B**) CAH as a function of Ar plasma treatment time on shrunk PO substrates. Error bars correspond to standard error, and the dashed lines correspond to superhydrophobicity.

To characterize the SH nature of the shrunk Ar-treated surfaces, CA and CAH measurements were conducted. Figure 3A presents CA measurements of water on the shrunk Ar-treated surfaces as measured by the DSA. Without any plasma treatment, the CA of the shrunk PO film is 102° ± 6°. As shown in Figure 3A, the CA was noted to increase when the surface was exposed to longer Ar plasma times. CA greater than 150°, which is characteristic of SH substrates, were found to occur on surfaces with an Ar exposure time of 30 min or higher (154° ± 6° for 30 min, 158° ± 4° for 40 min, and 158° ± 3° for 60 min). 

CAH measurements are presented in Figure 3B. The initial CAH for water on the untreated shrunk PO was 16° ± 2° and decreased steadily with longer Ar plasma exposure time. PO films treated with Ar plasma longer than 10 min have CAH values approaching 6° ± 1°. This low CAH indicates superhydrophobicity and correlates with the weak adhesion of fluid to the surface. 

As seen in Figure 2, the surface of the PO film becomes rougher with longer exposure to the Ar plasma. The observed nanoscale roughness for these longer exposure times correspond well with the SH nature of the surfaces. On such textured surfaces, the behavior of the water droplet can be explained through the lotus effect or the Cassie-Baxter model [12]. As the roughness of the structures and the density of air pockets increase, the CA increases and a low CAH is observed [37,38].

The combination of having CA greater than 150° and CAH less than 10° indicates a SH surface. Therefore, PO films with Ar plasma times greater than 30 min are characterized as SH.

### 3.3. Protein Capture and Detection

To illustrate the contrast in fluid behavior on a hydrophobic surface compared to a SH surface, hydrophilic anchor points of various sizes (2, 1, and 0.5 mm) were patterned onto non-Ar-treated shrunk surfaces and SH Ar-treated surfaces. The anchor points were created by exposing the samples to air plasma through a mask. Exposure to air plasma caused molecules from the air (such as oxygen) to bombard the surface with charged particles, resulting in a chemically-modified surface with an increased surface energy. Due to the short duration of the air plasma treatment (1 min), the air plasma treatment does not add structural modifications to the surface.

**Figure 4:**
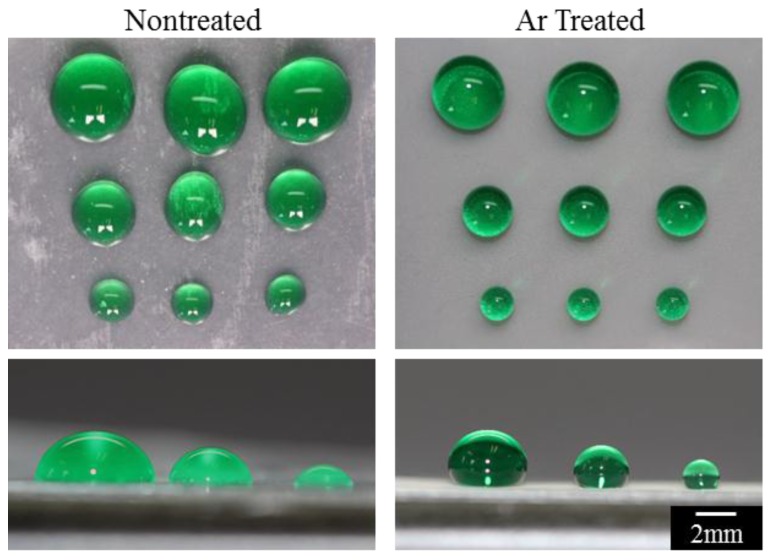
Air plasma was used to chemically pattern an array of hydrophilic anchor points (of sizes 2, 1, and 0.5 mm from top to bottom) from 2, 1, and 0.5 mm) on both non-treated and Ar-treated substrates. On the non-treated surface (left, top, and bottom) fluid (30, 15, and 5 µL) was not confined to the specified footprint. Conversely, on the SH Ar-treated surface (right, top, and bottom), the fluid remained within the patterned anchor point. The top images represent a top-down view of the droplets while the bottom images illustrate the side-view.

As shown in Figure 4, the fluid dispensed onto the hydrophilic anchor points on the non-Ar-treated surfaces and SH Ar-treated surfaces yielded different droplet shapes even with the same volumes of fluid (30, 15, 5 uL) added to the 2, 1, and 0.5 mm anchor points, respectively. On the non-Ar-treated surfaces, fluid overflowed from the patterned regions and was not confined by the specified hydrophilic footprint. Furthermore, the droplet shape was not symmetrical or controlled, and the spacing between spots was not uniform. In contrast, the fluid was precisely bound and confined to the defined spot on the SH Ar-treated surfaces. Droplets on the SH Ar-treated surfaces were semi-spherical with uniform spacing and had a consistent shape dependent on the patterned footprint and applied volume.

To demonstrate that these Ar-treated SH surfaces have the same utility as our previously fabricated SH surfaces, we applied a simple bioassay where we detected the presence of proteins in urine. A known advantage to SH surfaces is its ability to enhance detection of biomolecules in bodily fluids via simple evaporation of the fluid on the SH substrate [7]. To conduct this assay, fluid was confined by a hydrophilic anchor point, which was patterned onto the SH surface using air plasma treatment. As the fluid evaporates, the biomolecules contained within the droplet collapse and concentrate solely in the hydrophilic anchor point. On a flat substrate, molecules are not bound by the anchor point. 

**Figure 5:**
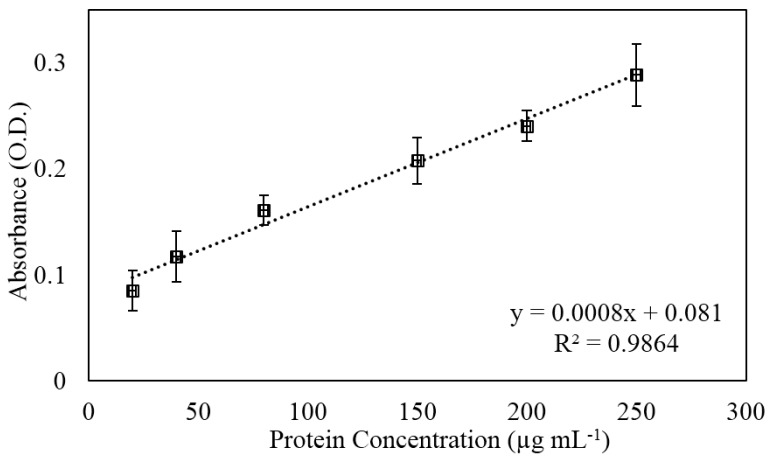
Urine spiked with BSA is detected by patterning the Ar plasma-treated samples for capture.

To determine feasibility of this phenomenon on the Ar-treated SH surfaces, we evaporated urine with varying protein concentrations onto a patterned array of hydrophilic spots. Upon addition of a protein assay dye reagent to the concentrated urine pellet, we were able to reliably detect physiologically-relevant ranges of protein (Figure 5) that were consistent with our previous findings our embossed SH surfaces. Thus, we confirmed that the Ar-treated SH substrates can be employed to enhance detection of biomolecules in a similar manner as our prior SH substrates. As these Ar-treated SH surfaces are created through a simpler fabrication approach than previously, we anticipate that these substrates will be readily adaptable to quickly developing simple, point-of-care diagnostic devices.

### 3.4. Open-Channel Microfluidic Wicking

In line with our interest for utilizing the Ar SH surfaces for point-of-care diagnostic applications, we have tested the ability to use these substrates for creating self-driven, open-channel microfluidic flow. Open-channel microfluidic assays with self-driven flow can be beneficial because they mitigate the need for bulky, external equipment such as pumps and tubing. Furthermore, open-channels can overcome many of the problems associated with closed-channel microfluidics, such as nonspecific sidewall adsorption, loss of reagents, clogging, and bubbles [39,40,41].

**Figure 6:**
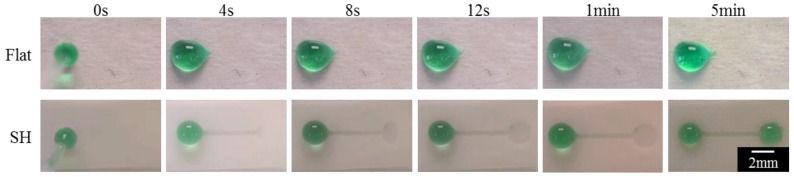
Superhydrophilic patterned channels allow fluid to wick along microchannels on the SH Ar-treated (bottom) samples but not along the flat, non-treated samples (top).

Self-driven flow on SH surfaces can be accomplished by patterning the surface with air plasma to achieve superhydrophilic channels. As described earlier in Section 3.3, the air plasma treatment causes a chemical modification to the surface through the addition of charged molecules. Coupled with the highly-structured topology of the Ar-treated SH surface, the chemical modification greatly increases the surface energy, causing a wetting transition from the Cassie–Baxter state to the Wenzel state and ultimately resulting in superhydrophilicity. The high differential in CA (>150° for SH and approaching 0° for superhydrophilic) between the patterned microchannel interface leads to fluid wicking along the channel [42,43,44]. As shown in Figure 6 (and Supplemental Videos 1 and 2), fluid flows along the 5 mm-long microchannel within 13 ± 4 s and reaches equilibrium within 5 min. In contrast, fluid does not flow along a similarly patterned microchannel on a non-SH surface because the CA differential is not great enough to drive fluid flow. Hence, we demonstrate feasibility of open-channel fluid wicking on the Ar-treated SH surfaces through simple fabrication methods.

## 4. Conclusions

In summary, we have demonstrated that Ar plasma can be used to etch pre-stressed PO films, thus creating a stiff layer that generates SH surfaces directly in the PO film upon shrinking. In contrast to previous studies, our method directly achieves SH surfaces without the need for post-processing and complex chemical additions. We further show that such SH surfaces can be selectively patterned by air plasma to create hydrophilic regions to capture protein in urine for molecular diagnostics. The contrast in wettability between the SH and superhydrophilic regime generated fluid flow along microchannels to achieve self-driven, open-channel microfluidics, negating the need for external equipment and eliminating the problems associated with closed-channel microfluidics. In particular, the ability to selectively pattern these SH surfaces through our method could be useful for low energy transport of fluids in molecular diagnostics, thus making our surfaces feasible for broad applications such as point-of-care diagnostics.

## Supplementary Materials

The following are available online at www.mdpi.com/1996-1944/9/3/196/s1. Figure S1: Microscale Roughness, Video S1: Wicking video on a flat patterned surface, Video S2: Wicking video on a SH patterened surafce.
