# Supplementary figures and images for: Nanotextured Shrink Wrap Superhydrophobic Surfaces by Argon Plasma Etching
Source: Materials (Basel). 2016 Mar 14;9(3):196. doi: 10.3390/ma9030196 (PMC5456652; doi:10.3390/ma9030196)

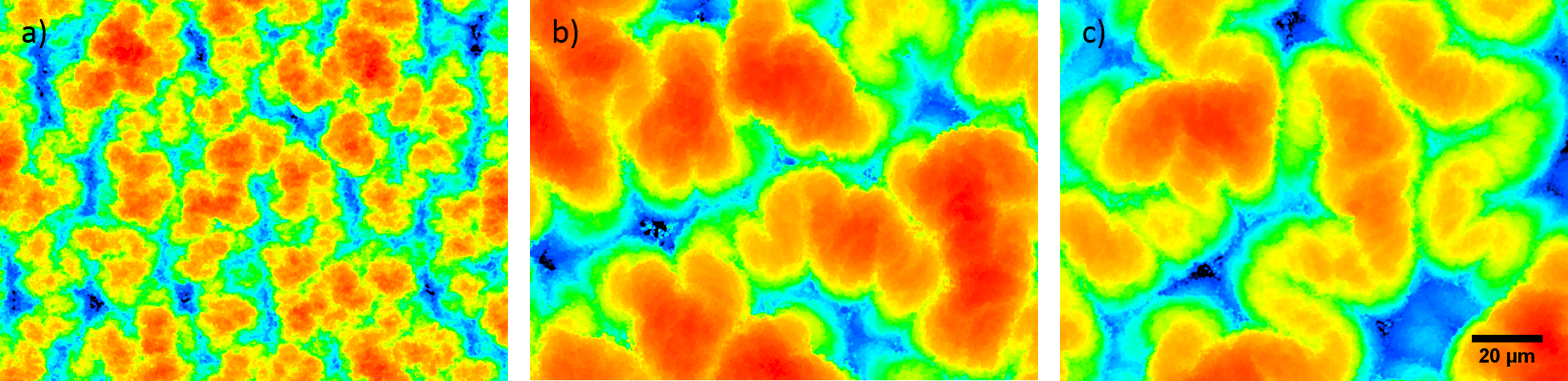

Supplement: Supplementary file 1 [file materials-09-00196-s001.zip › Supplemental Figure S1.png]
